# Synthesis of *N*-(Methoxycarbonylthienylmethyl)thioureas and Evaluation of Their Interaction with Inducible and Neuronal Nitric Oxide Synthase

**DOI:** 10.3390/molecules15053121

**Published:** 2010-04-28

**Authors:** Ghadeer A.R.Y. Suaifan, Claire L.M. Goodyer, Michael D. Threadgill

**Affiliations:** 1 Department of Pharmaceutical Sciences, Faculty of Pharmacy, The University of Jordan, Amman, Jordan; 2 Department of Pharmacy & Pharmacology, University of Bath, Claverton Down, Bath BA2 7AY, UK; E-Mail: M.D.Threadgill@bath.ac.uk (M.D.T.)

**Keywords:** nitric oxide synthase, iNOS, stimulation, thienylmethylthioureas, Nitric Oxide Synthase (NOS), endothelial Nitric Oxide Synthase (eNOS), neuronal Nitric Oxide Synthase (nNOS), inducible Nitric Oxide Synthase (iNOS), l-arginine (l-Arg)

## Abstract

Two isomeric *N*-(methoxycarbonylthienylmethyl)thioureas were synthesised by a sequence of radical bromination of methylthiophenecarboxylic esters, substitution with trifluoroacetamide anion, deprotection, formation of the corresponding isothiocyanates and addition of ammonia. The interaction of these new thiophene-based thioureas with inducible and neuronal nitric oxide synthase was evaluauted. These novel thienylmethylthioureas stimulated the activity of inducible Nitric Oxide Synthase (iNOS).

## 1. Introduction

Nitric oxide (•NO) is biosynthesised from l-arginine in two steps catalysed by nitric oxide synthase (NOSs) giving l-citrulline as a co-product. There are three isoforms, endothelial NOS (eNOS) and neuronal NOS (nNOS) (constitutive Ca^2+^-dependent forms) and an inducible form (iNOS). Underactivity and overactivity of each of these isoforms can be associated with disease states. Excessive NO production by eNOS within blood vessel walls is thought to be the basis for conditions such as septic- and cytokine-induced circulatory shock [1]. However, if too little NO is produced, this can lead to conditions such as high blood pressure, angina and impotence [2]. An overexpression of nNOS in circulating neutrophils has been found in patients with Parkinson’s disease [3] and nNOS activity is thought to be linked to migraine headaches. NO production by iNOS is essential for the defence mechanism of an organism; however, overactivity of iNOS has been related to several pathological conditions, including cancer, arthritis and diabetes [4–5]. Thus Selective inhibition of each of these three isoforms (eNOS, nNOS and iNOS) has potential applications in medicinal chemistry. Most potent inhibitors of NOS ligate to the haem iron at the active sites of the enzymes, through amidines and guanidines and through (iso)thioureas. Early inhibitors included close analogues of l-Arg, such as *N*^δ^-(iminoethyl)-l-ornithine (l-NIO) [6] and *N*^ω^-nitro-l-arginine methyl ester (l-NAME). [7] 

**Figure 1:**
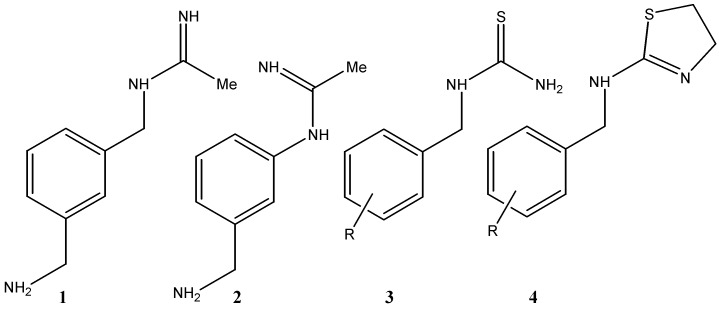
Structures of the iNOS-selective inhibitor 1400W **1**, the nNOS-selective lower homologue **2** and the iNOS stimulators **3** and **4**. R = 3-NH_2_, 4-NH_2=_, 3-CO_2_H, 4-CO_2_H.

More recent iNOS-selective inhibitors contain cyclic amidines to bind to the haem iron but lack the amino-acid motif of l-Arg [8,9]. Most interesting are 1400W (**1**) [10] (Figure 1), an *N*-benzylacetamidine which is a highly selective inhibitor of rat iNOS, and its lower homologue **2** [11] (Figure 1), an *N*-phenylacetamidine which is selective for inhibition of nNOS. 

We recently reported [12] a series of *N*-benzyl- and N-phenyl-thioureas and analogous 2-(substituted-amino)-4,5-dihydrothiazoles; several of these were moderate inhibitors of iNOS but were without activity against nNOS. More interestingly, *N*-benzylthioureas **3** (Figure 1) and 2-benzylamino-4,5-dihydrothiazoles **4** (Figure 1) caused significant stimulation of iNOS activity when the agents were added to the enzyme preparation simultaneously with the substrate. Therefore, in this work, our aim was to synthesis a new target in which benzene has been deplaced with the approximately isosteric thiophene. This rigidity introduced into the target is designed to maximise interaction and, more importantly, to differentiate between various NOS isoforms. Information from potent but non-isoform-selective NOS inhibitors suggested that 4,5-dihydrothiazole, thiourea and imidazole are good head groups for haem-iron-binding. So, This work extended our previous work into two exemplary *N*-thienylmethylthioureas.

## 2. Results and Discussion

**Scheme 1:**
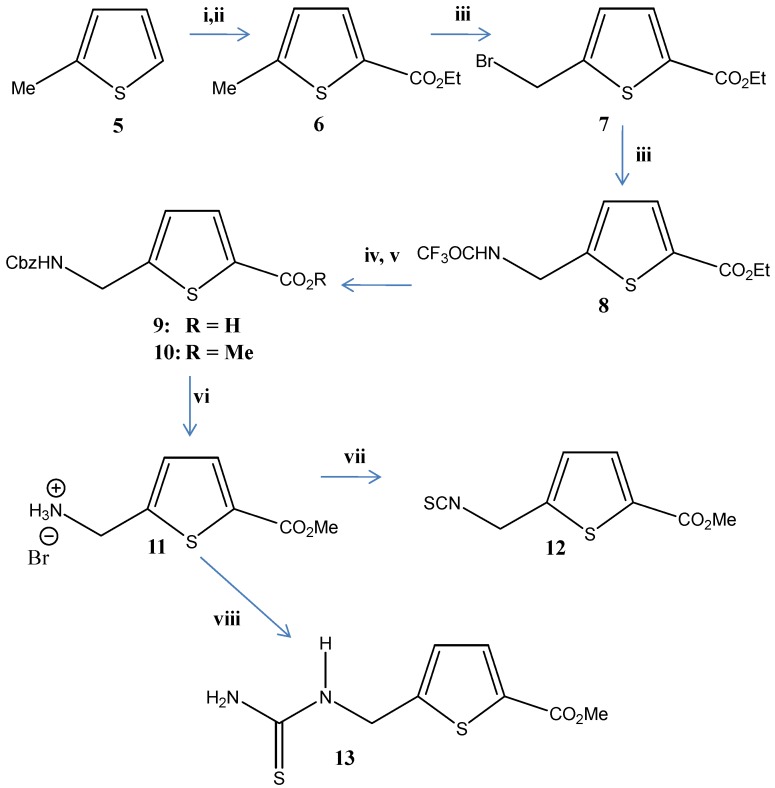
Synthetic route to target thioureidomethylthiophene **13**.

Retrosynthetic analysis suggested that the commercially available 2-methylthiophene (**5**) would be an ideal starting material. Abstraction of the relatively acidic thiophene 2-proton by butyl lithium would give an appropriate nucleophile for the reaction with a reactive carbonyl electrophile. Therefore, lithiation of **5** with butyl lithium at 0 °C, followed by quench with ethyl chloroformate (a reactive carboxy electrophile equivalent) gave the required ester **6** in 68% optimised yield (Scheme 1). 

A similar lithiation of **5** with butyl lithium, followed by quench with methyl chloroformate, afforded mixture of products. Thus the precise nature of the trapping electrophile was important. Bromination using *N*-bromosuccinimide (NBS) in the presence of perchloric acid introduced functionality to the methyl group, although the bromomethyl analogue **7** was obtained in only 7% isolated yield; ethyl 4-bromo-5-methylthiophene-2-carboxylate and ethyl 4-bromo-5-bromomethylthiophene-2-carboxylate were also identified in the reaction mixture. Thiourea is nucleophilic at sulfur; thus direct displacement of the bromine of **7** would give the isothiourea, rather than the target thiourea **13**, so the thiourea had to be introduced stepwise. Replacement of the bromine with the anion derived from trifluoroacetamide provided one nitrogen in **8**. From here, no conditions were found which would remove the trifluoroacetyl masking group from the primary amine while leaving the ester unscathed. Hence vigorous base-hydrolysis cleaved both the amide and the ester to give the highly polar 5-(aminomethyl)thiophene-2-carboxylate which could only be isolated from the reaction mixture after convertion in situ to the Cbz-protected derivative **9**. Now, the methyl ester **10** was formed and the Cbz was removed quantitatively and selectively, using hydrogen bromide, to afford **11**. From here, our previously developed method [12,13,14] was employed to convert the primary amine firstly to the isothiocyanate **12** (with thiophosgene) and then to the required thiourea **13**.

**Scheme 2:**
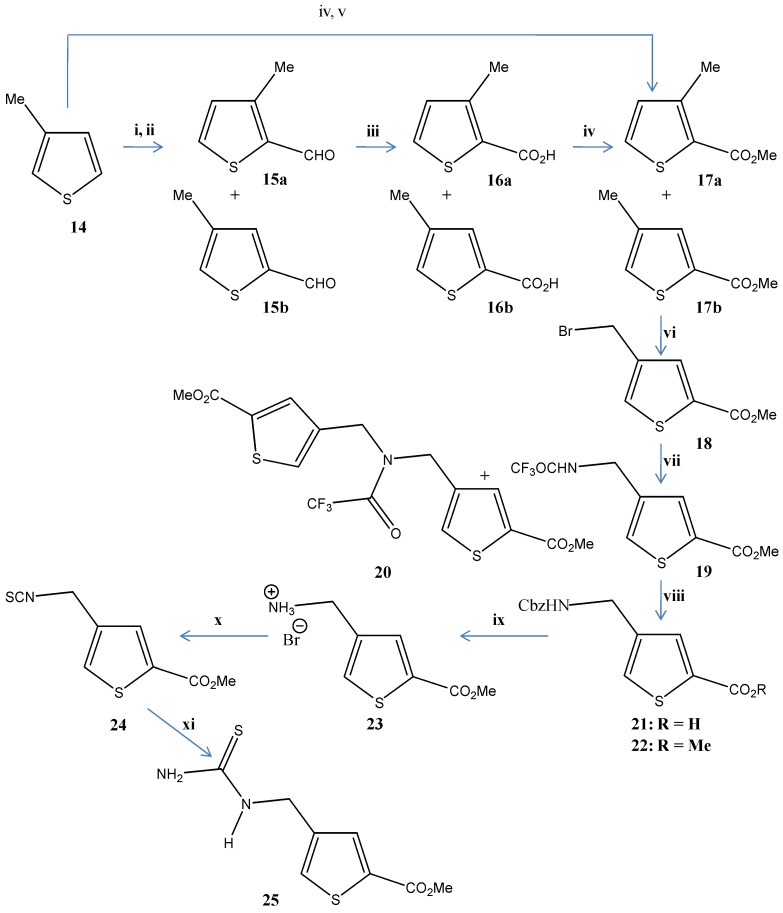
Synthetics route to target thioureidomethylthiophene **25**.

Scheme 2 shows the synthetic approaches to the 2,4-disubstituted target thiourea **25**. Attempted introduction of a carbonyl substituent to 3-methylthiophene **14**, by lithiation and reaction with dimethylformamide (DMF), gave an inseparable mixture of the regioisomeric aldehydes **15a**,**b**. Oxidation of this mixture to the carboxylic acids **16a**,**b** was achieved with silver(I) oxide but this also failed to allow isolation of the pure required regioisomer **16b**. Similarly, quench of the lithiated species derived from **14** with methyl chloroformate gave a 1:4 mixture of the esters **17a** and **17b**, respectively. Unfortunately, this mixture was again inseparable. Radical bromination of the mixture was achieved by treatment with one equivalent of NBS, using dibenzoyl peroxide as radical initiator. However, a mixture of unreacted starting material, ring-brominated products, and side-chain brominated (and polybrominated) products were produced. Repeated attempts using two equivalents of NBS gave higher percentages of ring-brominated products. Separation of the mixture *via* chromatography afforded 4.5% of **18**. As in the 2,5-disubstituted sequence, reaction with trifluoroacetamide anion gave a moderate yield of the secondary amide **19** but, despite the presence of a large excess of the nucleophile, a small quantity of the dialkylated product **20** was also isolated. Hydrolysis of the trifluoroacetamide and the ester functional groups of **19** was carried out simultaneously using sodium hydroxide. In the same reaction flask, a Schotten-Baumann reaction was carried out using benzyl chloroformate, giving, after acid work-up, the Cbz compound **21** in 82% yield. This was esterified with methanol and H_2_SO_4_ to give the ester **22** in high yield. As before, the protecting Cbz group of **22** was cleaved quantitatively upon treatment with hydrogen bromide in acetic acid. Compound **23** was then converted to the isothiocyanate **24** with thiophosgene in the presence of CaCO_3_. Reaction of **24** with ammonia gave the thiourea **25** in excellent yield.

### Biological Evaluation

Compounds **13** and **25** were evaluated for their effects on the activity of rat brain nNOS and of recombinant human iNOS (hiNOS) at a test concentration of 100 µM using the l-[U-^14^C]-arginine to l-[U-^14^C]-citrulline conversion assay. 1400W **1** [10] and *N*-(3-aminobenzyl)thiourea **3** (R = 3-NH_2_) [12] were used for comparison. Compounds were evaluated either by adding the radiolabelled substrate simultaneously with the inhibitor or with a pre-incubation of 10 min before the substrate was added. This pre-incubation has been reported to be optimum for the inhibitory activity of 1400W **1**[10]. This pre-incubation was investigated since test compounds can be considered to be analogues of the slow inhibitor 1400W **1**. 

As expected, 1400W **1** was a potent inhibitor of iNOS activity, both with and without pre-incubation with the enzyme. *N*-(3-Aminobenzyl)thiourea **3** was inactive towards the rat nNOS isoform but, as noted previously [12], stimulated formation of l-[U-^14^C]-citrulline from l-[U-^14^C]-arginine when the agent was added simultaneously with the substrate. The *N*-(thienylmethyl)thioureas **13** and **25** showed similar activity to that of **3**, stimulating human iNOS activity but not modulating the activity of this isoform at all when the compounds were pre-incubated with the enzyme before addition of the substrate. Neither **13** nor **25** affected the activity of rat nNOS, with or without pre-incubation. 

## 3. Experimental

### 3.1. General

NMR data were recorded on either JEOL/Varian GX 270 or EX 400 spectrometers, using solutions in deuteriochlorform (CDCl_3_), unless otherwise stated. IR spectra of samples were recorded as potassium bromide (KBr) discs, unless otherwise stated. Mass Spectra were recorded using a VG Analytical Mass Spectrometer in the FAB positive ion mode, unless otherwise stated. Elemental analyses was determined using Exeter Analytical Inc. CE-440 Elemental Analyzer. Solutions in organic solvents were dried with magnesium sulfate (MgSO_4_) and solvents were evaporated under reduced pressure. Experiments were conducted at ambient temperature, unless otherwise stated. Melting points were determined using a Reichert-Jung Thermo Galen Kofler block. 

### 3.2. Chemistry

*Ethyl 5-methylthiophene-2-carboxylate* (**6**). 2-Methylthiophene (**5**, 20.0 g, 204 mmol) was added to butyl lithium (220 mmol) in dry tetrahydrofuran (400 mL) at 0 °C. The mixture was stirred at 0 °C for 3 h before being added to ethyl chloroformate (28.2 g, 260 mmol) in tetrahydrofuran (200 mL) at 0 °C. The mixture was stirred at 20 °C for 16 h, and then poured onto ice. The organic layer was washed with water, hydrochloric acid (1 M), aq. sodium hydrogen carbonate and water. Drying, evaporation and distillation gave **6** (23.0 g, 68%) bp~_1_ 120°C (lit. [15] bp_5_ 87-89°C); ^1^H-NMR δ 1.33 (3 H, t, *J* = 7.0 Hz, CH_2_C*H*_3_), 2.48 (3 H, brs, 5-Me), 4.30 (2 H, q, *J* = 7.0 Hz, CH_2_), 6.72 (1 H, dq, *J* = 3.7, 1.2 Hz, 4-H), 7.57 (1 H, d, *J* = 3.7Hz, 3-H).

*Ethyl 5-bromomethylthiophene-2-carboxylate* (**7**). Compound **6** (1.00 g, 5.9 mmol) was stirred with N-bromosuccinimide (1.08 g, 6.1 mmol) and perchloric acid (60%, 30 μL) in hexane (3.0 mL) for 24 h. The evaporation residue, in EtOAc, was washed with saturated aq. sodium sulfite solution. Drying, evaporation and chromatography (hexane / EtOAc 9:1) gave **7** (160 mg, 11%) as a colourless oil: ^1^H-NMR δ 1.37 (3 H, t, *J* = 7.0 Hz, Me), 4.35 (2 H, q, *J* = 7.0 Hz, OCH_2_), 4.68 (2 H, s, CH_2_Br), 7.08 (1 H, dt, *J* = 3.9, 0.8 Hz, 4-H), 7.63 (1 H, d, *J* = 3.9 Hz, 3-H); MS *m/z* 251/249 (M + H), 170 (M + H – Br); Found C, 38.60; H, 3.77; C_8_H_9_BrO_2_S requires C, 38.55; H, 3.64%.

*Ethyl 5-(trifluoroacetamidomethyl)thiophene-2-carboxylate* (**8**). Trifluoroacetamide (410 mg, 3.6 mmol) was stirred with potassium *t*-butoxide (410 mg, 3.6 mmol) in dry tetrahydrofuran (4 mL) for 1 h. Compound **7** (130 mg, 520 μmol) in dry tetrahydrofuran (3 mL) was added and the mixture was stirred for 15 h. The evaporation residue, in dichloromethane, was washed with water, hydrochloric acid (1 M) and water. Drying and evaporation gave **8** (90 mg, 62%) as a pale yellow solid: mp 65-66 °C: IR ν_max_ 3333, 1719, 1683 cm^-1^; ^1^H-NMR δ 1.37 (3 H, t, *J* = 7.0 Hz, Me), 4.34 (2 H, q, *J* = 7.0 Hz, OCH_2_), 4.72 (2 H, d, *J* = 5.9 Hz, CH_2_N), 7.03 (1 H, dt, *J* = 3.9, 0.8 Hz, 4-H), 7.67 (1 H, d, *J* = 3.5 Hz, 3-H); NMR δ_F_ -76.2 (3 F, s, CF_3_); MS *m/z* 282.0413 (M + H) (C_10_H_11_F_3_NO_3_S requires 282.0412); Found C, 42.90; H, 3.49; N, 4.98; C_10_H_10_F_3_NO_3_S requires C, 42.68; H, 3.59; N, 4.98%. 

*5-(Benzyloxycarbonylaminomethyl)thiophene-2-carboxylic acid* (**9**). Compound **8** (3.50 g, 12.0 mmol) was boiled under reflux with sodium hydroxide (3.93 g, 98 mmol) in water (50 mL) and methanol (50 mL) for 16 h. The methanol was evaporated. Benzyl chloroformate (5.1 mL, 36 mmol) was added at 0 °C and the mixture was stirred vigorously for 4 h. The mixture was washed with diethyl ether. The aqueous layer was acidified (hydrochloric acid) and extracted with ethyl acetate. Drying and evaporation gave **9** (3.33 g, 92%) as a yellow solid: mp 142-145 °C; IR ν_max_ 3344, 2700, 1694, 1547, 1528 cm^-1^; ^1^H-NMR [(CD_3_)_2_SO] δ 4.36 (2 H, d, *J* = 6.2 Hz, CH_2_N), 5.04 (2 H, s, CH_2_O), 6.98 (1 H, d, *J* = 3.5 Hz, 4-H), 7.3 (5 H, m, Ph-H_5_), 7.55 (1 H, d, *J* = 3.5 Hz, 3-H), 8.02 (1 H, t, *J* = 6 Hz, NH); MS *m/z* 292.0644 (M + H) (C_14_H_14_NO_4_S requires 292.0640); Found: C, 57.50; H, 4.41; N, 4.63; C_14_H_13_NO_4_S requires C, 57.70; H, 4.50; N, 4.81%.

*Methyl 5-(benzyloxycarbonylaminomethyl)thiophene-2-carboxylate* (**10**). Compound **9** (500 mg, 1.7 mmol) was boiled under reflux with sulfuric acid (1.0 mL) in methanol (50 mL) for 2 d. Aq. sodium hydrogen carbonate was added until no further bubbling occurred. The suspension was filtered. The evaporation residue was washed with water and dried to afford **10** (460 mg, 88%) as a white solid: mp 80-83 °C; ^1^H-NMR δ 3.87 (3 H, s, Me), 4.55 (2 H, d, *J* = 5.9 Hz, CH_2_N), 5.14 (2 H, s, CH_2_O), 5.23 (1 H, br, NH), 6.95 (1 H, d, *J* = 3.5 Hz, 4-H), 7.31-7.36 (5 H, m, Ph-H_5_), 7.64 (1 H, d, *J* = 3.5 Hz, 3-H); MS *m/z* 398 (M + glycerol + H), 306.0813 (M + H) (C_15_H_16_NO_4_S requires 306.0800), 259 (M - CO_2_Me); Found C, 58.99; H, 5.00; N, 4.52; C_15_H_15_NO_4_S requires C, 58.98; H, 4.95; N, 4.59%.

*Methyl 5-(aminomethyl)thiophene-2-carboxylate hydrobromide* (**11**). Compound **10** (100 mg, 330 μmol) was stirred with hydrogen bromide in AcOH (15%, 2.0 mL) for 20 min. The evaporation residue was washed (10 ×) with diethyl ether and dried to afford **11** (83 mg, quant.) as a white solid: mp 212-215 °C; ^1^H-NMR [(CD_3_)_2_SO] δ 3.83 (3 H, s, Me), 4.31 (2 H, s, CH_2_), 7.30 (1 H, d, *J* = 3.9 Hz, 4-H), 7.76 (1 H, d, *J* = 3.9 Hz, 3-H), 8.26 (3 H, br, N^+^H_3_); MS *m/z* 325 (M + mNBA + H), 172.0437 (M + H) (C_7_H_10_NO_2_S requires 172.0432); Found H, 4.01; N, 5.56; C_7_H_9_NO_2_S requires H, 4.00; N, 5.56%.

*Methyl 5-(isothiocyanatomethyl)thiophene-2-carboxylate* (**12**). Compound **11** (210 mg, 830 μmol) was stirred with calcium carbonate (90 mg, 870 μmol) and thiophosgene (200 mg, 1.7 mmol) in water (1.5 mL) and chloroform (10 mL) for 20 h. The solvent was evaporated from the organic layer. Chromatography (hexane / ethyl acetate 7:3) gave **12** (110 mg, 62%) as a colourless oil: IR (film) ν_max_ 2074, 1711 cm^-1^; ^1^H-NMR δ 3.87 (3 H, s, Me), 4.85 (2 H, s, CH_2_), 7.03 (1 H, dt, *J* = 3.9, 0.8 Hz, 4-H), 7.76 (1 H, d, *J* = 3.9 Hz, 3-H); MS *m/z* 367 (M + mNBA + H), 214.0003 (M + H) (C_8_H_8_NO_2_S_2_ requires 213.9997), 182 (M - S); Found C, 44.90; H, 3.32; N, 6.55. C_8_H_7_NO_2_S_2_ requires C, 45.05; H, 3.31; N, 6.57%.

*Methyl 5-(thioureidomethyl)thiophene-2-carboxylate* (**13**). Ammonia was passed through **12** (60 mg, 280 μmol) in dichloromethane at 0°C for 20 min. The mixture was stirred for 3 h at 0 °C. Evaporation gave **13** (65 mg, quant.) as a white solid: mp 131-132 °C: IR ν_max_ 3398, 1703, 1277 cm^-1^; ^1^H-NMR (CD_3_OD) δ 3.84 (3 H, s, Me), 4.93 (2 H, d, *J* = 5.9 Hz, CH_2_N), 7.06 (1 H, dt, *J* = 3.7, 0.7 Hz, 4-H), 7.65 (1 H, d, *J* = 4.0 Hz, 3-H); MS *m/z* 231.0255 (M + H) (C_8_H_11_N_2_OS_2_ requires 231.0262); Found C, 41.50; H, 4.32; N, 12.06; C_15_H_15_NO_4_S requires C, 41.72; H, 4.38; N, 12.16%.

*3-Methylthiophene-2-carboxaldehyde (15a) and 4-methylthiophene-2-carboxaldehyde* (**15b**). 3-Methylthiophene **18** (10.0 g, 102 mmol) was added slowly to butyl lithium (2.0 M in hexane, 55 mL, 110 mmol) in dry diethyl ether (175 mL) at 0 °C and the mixture was stirred for 3 h at 0 °C, before being added slowly to dimethylformamide (10.2 g, 140 mmol) in dry diethyl ether (35 mL) at 0 °C. The mixture was stirred for 16 h and poured onto ice. The organic layer was washed with water, hydrochloric acid (1 M), aq. sodium hydrogen carbonate and water. Drying, evaporation and distillation (*ca*. 1 torr) gave a mixture of **15a** and **15b** (12.6 g, 61%) as a colourless liquid: ^1^H-NMR δ 2.32 [2.3 H, s, Me (**15b**)], 2.58 [0.7 H, s, Me (**15a**)], 6.97 [0.22 H, d, *J* = 5.2 Hz, 4-H (**15a**)], 7.36 [0.78 H, d, *J* = 1.3 Hz, 5-H (**15b**)], 7.58 [0.78 H, d, *J* = 1.3 Hz, 3-H (**15b**)], 7.63 [0.22 H, d, *J* = 5.2 Hz, 5-H (**15a**)], 9.86 [0.78 H, d, *J* = 1.3 Hz, CHO (**15b**)], 10.04 [0.22 H, d, *J* = 1.3 Hz, CHO (**15a**)].

*3-Methylthiophene-2-carboxylic acid (16a) and 4-methylthiophene-2-carboxylic acid* (**16b**). Silver(I) oxide (4.6 g, 20 mmol) was stirred with mixture **19a**,**b** (2.5 g, 20 mmol) and sodium hydroxide (3.2 g, 80 mmol) in water (50 mL) for 16 h. The mixture was filtered, acidified (hydrochloric acid) and extracted with dichloromethane. Drying, evaporation and recrystallisation (hexane) gave a mixture of **16a** and **16b** (2.30 g, 82%) as white crystals: mp 118-120 °C. ^1^H-NMR δ 2.30 [2.7 H, s, Me (**16b**)], 2.57 [0.3 H, s, Me (**16a**)], 6.95 [0.1 H, d, *J* = 4.7 Hz, 4-H (**16a**)], 7.13 [0.9 H, m, 5-H (**16b**)], 7.48 [1 H, d, *J* = 5.1 Hz, 5-H (**16a**)], 7.69 [0.9 H, d, *J* = 1.6 Hz, 3-H (**16b**)]; MS *m/z* 143.0169 (M + H) (C_6_H_7_O_2_S requires 143.0167).

*Methyl 3-methylthiophene-2-carboxylate (17a) and methyl 4-methylthiophene-2-carboxylate* (**17b**). 3-Methylthiophene **14** (5.0 g, 51 mmol) was added slowly to butyl lithium (2.0 M in tetrahydrofuran, 28 mL, 56 mmol) in dry tetrahydrofuran (100 mL) at 0 °C. The mixture was stirred for 3 h at 0 °C, before being added to methyl chloroformate (6.33 g, 67 mmol) in dry tetrahydrofuran (20 mL) at 0 °C. The mixture was stirred for 16 h and poured onto ice. The organic layer was washed with water, hydrochloric acid (1 M), aq. sodium hydrogen carbonate and water. Drying, evaporation and distillation (*ca*. 1 torr) gave a mixture of **17a** and **17b** (5.00 g, 62%) as a colourless oil: ^1^H-NMR δ 2.28 [2.4 H, s, Me (**17b**)], 2.56 [0.6 H, s, Me (**17a**)], 3.86 (3 H, s, OMe), 6.91 [0.2 H, d, *J* = 5.1 Hz, 4-H (**17a**)], 7.13 [0.8 H, m, 5-H (**17b**)], 7.38 [0.2 H, d, *J* = 5.1 Hz, 5-H (**17a**)], 7.60 [1 H, d, *J* = 1.6 Hz, 3-H (**17b**)].

*Methyl 4-bromomethylthiophene-2-carboxylate* (**18**). Mixture **17a**,**b** (2.48 g, 16 mmol) was boiled under reflux with dibenzoyl peroxide (80 mg, 330 μmol) and N-bromosuccinimide (2.83 g, 16 mmol) in tetrachloromethane (40 mL) for 17 h. Additional dibenzoyl peroxide (50 mg, 210 μmol) was added every 10 min for 1 h and then every 30 min (twice). Evaporation and chromatography (hexane/ethyl acetate 9:1) gave **18** (170 mg, 5%) as a pale buff oil; ^1^H-NMR δ 3.89 (3 H, Me), 4.46 (2 H, s, CH_2_), 7.49 (1 H, d, *J* = 1.6 Hz, 5-H), 7.80 (1 H, d, *J* = 1.6 Hz, 3-H).

*Methyl 4-(trifluoroacetamidomethyl)thiophene-2-carboxylate (19) and N,N-bis((2-methoxycarbonyl-thien-4-yl)methyl)trifluoroacetamide* (**20**). Trifluoroacetamide (570 mg, 5.1 mmol) in dry THF (2 mL) was stirred with KOBu^t^ (570 mg, 5.1 mmol) in dry THF (2 mL) for 1 h. Compound **18** (170 mg, 0.72 mmol) in dry THF (2 mL) was added slowly and the mixture was stirred for 15 h. The evaporation residue, in CH_2_Cl_2_, was washed with H_2_O, aq. HCl (1 M) and H_2_O. Drying, evaporation and chromatography (CHCl_3_ / EtOAc 19:1) afforded **20** (10 g, 5%) as a yellow solid: IR (film) ν_max_ (NH), 1711 (CF_3_C=O), 1603 (amide I), 1541 (amide II) cm^-1^; ^1^H-NMR δ 3.89 (3 H, s, MeCO), 3.90 (3 H, s, MeCO), 4.50 (2 H, s, CH_2_), 4.54 (2 H, s, CH_2_), 7.34 (1 H, brs, 5-H), 7.36 (1 H, brs, 5-H), 7.57 (1 H, d, J = 1.6 Hz, 3-H), 7.60 (1 H, d, J = 1.6 Hz, 3-H); ^19^F-NMR δ -68.67 (3 F, s, CF_3_); MS m/z 422.0340 (M + H) (C_16_ H_15_F_3_NO_5_S_2_ requires 422.0344). Further elution gave **19** (70 mg, 35%) as a yellow solid: mp 80-83°C; IR ν_max_ 3305 (NH), 1703 (CF_3_C=O), 1562 (amide) cm^-1^; ^1^H-NMR δ 3.89 (3 H, s, Me), 4.53 (2 H, d, J = 5.8 Hz, CH_2_), 6.61 (1 H, br, NH), 7.45 (1 H, m, 5-H), 7.71 (1 H, d, J = 1.7 Hz, 3-H); ^19^F-NMR δ -76.2 (3 F, s, CF_3_); MS m/z 268.0255 (M + H) (C_9_H_9_O_3_F_3_NS requires 268.0246).

*4-(Benzyloxycarbonylaminomethyl)thiophene-2-carboxylic acid* (**21**). Compound **19** (200 mg, 0.75 mmol) was boiled under reflux with NaOH (300 mg, 7.5 mmol) in H_2_O (3 mL) and MeOH (3 mL) for 16 h. The MeOH was evaporated. Benzyl chloroformate (380 mg, 2.2 mmol) was added at 0 °C and the mixture was stirred vigorously for 4 h, before being washed with EtOAc. The aqueous layer was acidified (aq. HCl) and extracted with EtOAc. Drying, evaporation and chromatography (EtOAc) afforded **21** (180 mg, 82%) as a white solid: mp 149-151 °C; ^1^H-NMR [(CD_3_)_2_SO] δ 4.08 (2 H, d, *J* = 5.5 Hz, CH_2_N), 5.02 (2 H, s, CH_2_O), 7.33-7.36 (5 H, m, Ph-H_5_), 7.57 (1 H, m, 5-H), 7.60 (1 H, d, *J* = 1.2 Hz, 3-H), 7.82 (1 H, t, *J* = 5.9 Hz, NH); MS *m/z* 315.0503 (M + Na) (^13^C_1_^12^C_13_H_13_NNaO_4_S requires 315.0496), 314.0462 (M + Na) (^12^C_14_H_13_NNaO_4_S requires 3140463).

*Methyl 4-(benzyloxycarbonylaminomethyl)thiophene-2-carboxylate* (**22**). Compound **21** (500 mg, 1.7 mmol) was boiled under reflux with H_2_SO_4_ (1 mL) and MeOH (50 mL) for 2 d. Aq. NaHCO_3_ was added until no further bubbling occurred. The evaporation residue, in CHCl_3_ was washed with H_2_O. Drying and evaporation afforded **22** (430 mg, 82%) as a solid: 64-65ºC; ^1^H-NMR δ 3.88 (3 H, s, Me), 4.35 (2 H, d, *J* = 5.9 Hz, CH_2_N), 5.13 (3 H, br, CH_2_O + NH), 7.34-7.38 (6 H, m, Ph-H_5_ +5-H), 7.71 (1 H, br, 3-H); MS *m/z* 306.0809 (M + H) (C_15_H_16_NO_4_S requires 306.0800).

*Methyl 4-(aminomethyl)thiophene-2-carboxylate hydrobromide* (**23**). Compound **22** (280 mg, 0.92 mmol) in AcOH (2.8 mL) was stirred with 30% HBr in AcOH (2.8 mL) for 20 min. The evaporation residue was washed with Et_2_O (10 ×) and dried to afford **23** (230 mg, quant.) as a white solid: mp 203-205 °C; NMR ((CD_3_)_2_SO) δ_Η_ 3.85 (3 H, s, Me), 4.08 (2 H, s, CH_2_), 7.95 (1 H, d, *J* = 1.6 Hz, 5-H), 7.97 (1 H, m, 3-H), 8.13 (3 H, br, N^+^H_3_); MS *m/z* 172.0439 (M + H) C_7_H_10_NO_2_S requires 172.0432); Found C, 33.20; H, 3.88; N, 5.44; C_7_H_9_NO_2_S·HBr requires C, 33.33; H, 4.00; N, 5.56%.

*Methyl 4-(isothiocyanatomethyl)thiophene-2-carboxylate* (**24**). Compound **23** (200 mg, 0.8 mmol) was stirred with CaCO_3_ (90 mg, 0.93 mmol) and thiophosgene (180 mg, 1.6 mmol) in H_2_O (2.0 mL) and CHCl_3_ (10 mL) for 20 h. The solvent was evaporated from the organic layer. Chromatography (CH_2_Cl_2_) afford **24** (104 mg, 62%) as a white solid: mp 69-70 °C: IR ν_max_ 2115 (NCS), 1711 (C=O) cm^-1^; ^1^H-NMR δ 3.90 (3 H, s, Me), 4.71 (2 H, s, CH_2_), 7.48 (1 H, dt, *J* = 2.0, 0.8 Hz, 5-H), 7.75 (1 H, d, J = 2.0 Hz, 3-H); MS *m/z* 214 (M + H); Found C, 45.30; H, 3.37; N, 6.66. C_8_H_7_NO_2_S_2_ requires C, 45.03; H, 3.31; N, 6.57%.

*Methyl 4-(thioureidomethyl)thiophene-2-carboxylate* (**25**). NH_3_ was bubbled through **24** (60 mg, 0.28 mmol) in CH_2_Cl_2_ (3 mL) at 0°C for 20 min. The mixture was stirred for 3 hr at 0 °C. Evaporation afforded **25** (61 mg, 95%.) as a white solid: mp 131-132 °C: ^1^H-NMR δ 3.87 (3 H, s, Me), 4.64 (2 H, br, CH_2_), 5.98 (2 H, br, NH_2_), 6.77 (1 H, br, NH), 7.46 (1 H, br, 5-H), 7.72 (1 H, d, *J* = 1.6 Hz, 3-H); MS *m/z* 231.0272 (M + H) C_8_H_11_N_2_O_2_S_2_ requires 231.0262); Found C, 41.39; H, 4.29; N, 11.96; C_15_H_15_NO_4_S requires C, 41.72; H, 4.38; N, 12.16%.

### 3.3. Enzyme study

Measurements of the inhibitory activity of the test compounds against nNOS was perfomed using an enzyme preparation from rat brain (in which the large majority of the NOS activity is nNOS), whereas the assay of activity against iNOS was performed using preparation of recombinant human iNOS (hiNOS) overexpressed in an HT1080 cell line as described previously by us [12]. 

The assay used the conversion of l-[U-^14^C]-arginine to l-[U-^14^C]-citrulline as described previously by us [16]. Compounds were evaluated at 100 μM and assays were performed in two modes, simultaneous addition of the test compound to the enzyme preparation and of l-[U-^14^C]-arginine (to start the enzymic reaction) and pre-incubation of the test compound with the enzyme preparation for 10 min before initiation of the enzymic reaction by addition of l-[U-^14^C]-arginine. 

Briefly, for the studies with human iNOS, the enzyme was prepared as follows: an optimised mammalian expression vector (pEFIRES-P, courtesy of Dr. S. Hobbs, Cancer Research UK Centre for Cancer Therapeutics, ICR, London, U.K.) was designed to express human iNOS cDNA (courtesy of Prof. Ian Charles, University of London, London, U.K.). Expression was linked with the selectable marker gene (pac) at the level of mRNA and antibiotic selection (puromycin) directly enforced expression of the cDNA. This vector was been used to transfect the human fibrosarcoma cell line, HT1080 and a series of iNOSexpressing clones were produced. To avoid loss of viability through the cytotoxic consequences of excessive NO production, clones were grown in the presence of a non-toxic dose of *N*^G^-nitroarginine (100 mM). Routinely, clones were grown for 48 h in the absence of puromycin and *N*^G^-nitroarginine prior to extracting the iNOS enzyme. Cells were grown to near confluence and were harvested by trypsinisation. Cells were then washed twice in cold phosphate-buffered saline and homogenised in five volumes of ice-cold buffer containing HEPES (10 mM, pH 7.4), sucrose (320 mM), EDTA (100 mM), dithiothreitol (50 mM), leupeptin (10 mg mL^-1^), soybean trypsin inhibitor (10 mg mL^-1^) and aprotinin (2 mg mL^-1^). The preparations were then sonicated using an MSE Soniprep 150 for 35 s at a nominal frequency of 23KHz and oscillation amplitude between 5 and 10 mm. Samples were placed in ice between each sonication. These suspensions were allowed to stand in ice for a further 10 min, then centrifuged at 9,000g for 15 min at 4 °C. The post-mitochondrial supernatant was treated with Dowex-50W [(200–400), 8% cross-linked, Na^+^ form] to remove endogenous arginine. The supernatant was incubated with the resin for 5 min and centrifuged at 10,000 rpm for 5 min to pellet the resin. This process was repeated twice, after which the cytosol was treated as free of endogenous arginine and was used for assays of inhibition, using the usual protocol with and without pre-incubation of the test compounds with the preparation. The results are shown in Table 1 as the mean of triplicate experiments SEM.

## 4. Conclusions

In this work, we have extended the range of compounds that cause this apparent stimulation of iNOS activity from benzylthioureas [12] into thienylmethylthioureas. These results are significant and will be interesting for many scientists. 

Actually, the biochemical origin and mechanism of this stimulation is unclear, although a similar but weaker stimulation was reported by Ulhaq *et al*. for *S*-2-amino-5-(3-nitro-1,2,4-triazol-1-yl)pentanoic acid, *S*-2-amino-5-(3-amino-1,2,4-triazol-1-yl)pentanoic acid and *S*-2-amino-5-(2-cyanoimidazol-1-yl)pentanoate on iNOS and nNOS [16]. On the other hand, it has been reported that 3-phenyl-3,4-dihydro-1-isoquinolinamine is a weak inhibitor of iNOS and nNOS [17]. However, analogous 6-phenyl-4-amino-6,7-dihydrothienopyridines, in which the benzo ring has been replaced by a thieno ring fusion, are potent iNOS and nNOS inhibitors [18].

Several synthetic guanidines and *N*-hydroxyguanidines can act as artificial substrates for nitric oxide synthases [19–20]. However, since the assay used by us does not measure nitric oxide production but rather measures conversion of radiolabelled l-Arg to radiolabelled l-Cit, it is clear that the increased activity of the iNOS is a genuine stimulation. Therefore, one might speculate that these compounds interact with the enzyme through more than one binding site under different conditions; binding to one site would have a stimulatory effect while binding to the other would have inhibitory effect. The binding to the allosteric stimulatory site may be fast, whereas inhibitory binding of some known inhibitors (e.g. 1400W) to the catalytic haem site is known to be slow [10].

Recently, it was demonstrated that 1400W is not a slow-binding inhibitor but a time-dependent inactivator of iNOS [22]. Therefore our result could be also explained as follows: Binding is rapid, but initially reversible; the rate-determining step is inactivation of the enzyme by heme modification. Therefore, the "loss" of stimulation after 10 minutes is because the enzyme is becoming inactivated. This would then explain the stimulation effects reported without preincubation. 

Although most effort in this area has gone into inhibiting biosynthesis of •NO, increasing biosynthesis may have some therapeutic value. Nitric oxide blocks the expression of NADP oxidase, an enzyme important in the etiology of cardiovascular disease [23]. Also, sildenafil augments •NO concentrations by inhibiting phosphodiesterase-5 (PDE-5) and is useful in treating erectile disfunction [24] and, possibly, pulmonary hypertension and acute respiratory distress syndrome (ARDS) [25]. Direct stimulation of NOS isoforms may provide an alternative approach to these beneficial effects. 

Also, the thienylmethylthioureas and benzylthioureas reported here and previously [12] will be used in a future work to provide a pharmacophore from which more potent and more site-selective ligands may be designed by modeling and docking studies.

## Figures and Tables

*Reagents*: i, BuLi, THF, ii, EtO_2_CCl, THF; iii, NBS, HCIO_4_, hexane; iv, CF_3_CONH_2_, KOBu*^t^*, THF; v, NaOH, aq.MeOH, ∆; vi, MeOH, H_2_SO_4_; vii, HBr, HOAc; viii, CSCI_2_, CaCO_3_, H_2_O, CHCI_3_; ix, NH_3_, CH_2_CI_2__._

*Reagents*: i, BuLi, THF, ii, DMF, Et_2_O; iii, Ag_2_O, NaOH, H_2_O; iv, MeOH, H_2_SO_4_; v, EtO_2_CCI, THF; vi, NBS, (PhCO_2_)_2_, CCI_4_; vii, CF_3_CONH_2_, KOBu*^t^*, THF; viii, NaOH, aq.MeOH, ∆; ix, MeOH, H_2_SO_4_; x, HBr, HOAc; xi, CSCI_2_, CaCO_3_, H_2_O, CHCI_3_; xii, NH_3_, CH_2_CI_2_

**Table 1:** Stimulation of human iNOS and rat nNOS activity by 1400W **1**, *N*-(3-aminobenzyl)thiourea **3** and the *N*-thienylmethylthioureas **13** and **25**.

Compound	% Stimulation of hiNOS activity ^a^		IC50 (µM) hiNOS	% Stimulation of nNOS activity^a^	
	t = 0 min ^b^	t = 10 min ^b^	t = 15 min ^b^	t = 0 min ^b^	t = 10 min ^b^
**1**	-79 ± 1	-82 ± 1	<4	ND	ND
**3** (R = 3-NH_2_)	+58 ± 1	+9 ± 1		+1 ± 6	-9 ± 6
**13**	+62 ± 2	-4 ± 3		-1 ± 4	+3 ± 3
**25**	+37 ± 27	-8 ± 11		+6 ± 1	+6 ± 7

^a^ Concentration of test compound 100 μM; ^b^ t refers to the time between addition of the test compound and addition of l-[U-^14^C]-arginine to initiate the enzymic reaction.
